# Permanent Paralysis of One Side of Face, from Disease about the Angle of the Jaw

**Published:** 1833

**Authors:** 


### Art. VIII. Permanent Paralysis of one side of the Face, from disease about the angle of the Jaw.

Four years ago, we had an opportunity of collecting the following facts, from conversation with the patient, the Rev. H. C. T., of the State of Virginia.

Before he was quite a year old, several large boils or abscesses, the eschars of which still remain, formed behind, over, and in front of the angle of the lower jaw of the right side. The discharge from them continued for several months, and on ceasing, the left angle of his mouth was found to be drawn backwards. The opposite side, in fact, was partially paralysed, and has continued in that state ever since, a period of 34 years. He grew up a healthy and vigorous man, and has generally been very well. The right side of the forehead is smooth, and does not contract under the influence of the will; nor does the brow ascend, when he attempts to throw the occipito-frontalis into action. The right *posterior* extremity of that muscle, however, acts as perfectly as the left. The margin of the right upper eyelid, crosses the globe in the usual place; but that of the lower, is a little depressed. He cannot draw it up to the usual line, in attempting to close the eye, but can depress the other so as to cover about two-thirds of the open space between them. The whole right cheek is smooth and spread out. He cannot expand the nostril of that side, and the depression above the cartilage is wanting. By an effort, he can draw snuff into that nostril; but less air passes through it, in ordinary, than through the other nostril. He can neither raise, depress, nor project the right angle of his mouth. The right brow has less hair, and is more flatted than the left; the frontal sinus is quite undeveloped; that of the other side, sufficiently prominent. All the movements of the eyeball are perfect; the vision of the eye is good, and in harmony with the other; the the eye is not inflamed, but a little watery. His mastication on that side is strong and perfect. His tongue straight when projected, and of the usual form, size, and capabilities. The taste and smell of the affected side, are like those of the other. The sensibility of the skin of that side, to cold and mechanical irritation, together with the colour, are natural. His voice is somewhat affected; but still he is not an unimpressive public speaker to the ear, though the movements of his face on such occasions are unpleasant to the eye.

The gentleman, of whose case I have given an account, applied to us for advice, but we could afford him none. It seemed quite obvious, that the portio dura, (facial respiratory nerve of Bell,) had suffered a permanent lesion, in his infancy, and that no remedy existed. Had this occurred after he had learned to talk, it is probable his speech would have been much more affected than it is. Trial and continued effort at imitation, would of course enable him to surmount the difficulties which were thrown in his way, at the early period in which he suffered the injury. *Perhaps* the trifacial nerve of that side gradually took on some degree of vicarious function, in reference to speech. It is worthy of remark, that the development of the os frontis of that side was affected by this injury.

